# Extracting Patient-Centered Outcomes from Clinical Notes in Electronic Health Records: Assessment of Urinary Incontinence After Radical Prostatectomy

**DOI:** 10.5334/egems.297

**Published:** 2019-08-20

**Authors:** Davide Gori, Imon Banerjee, Benjamin I. Chung, Michelle Ferrari, Paola Rucci, Douglas W. Blayney, James D. Brooks, Tina Hernandez-Boussard

**Affiliations:** 1 University of Bologna, IT; 2 Stanford University, US

**Keywords:** patient-reported outcomes, urinary incontinence, electronic health records, patient surveys

## Abstract

**Objective:**

To assess documentation of urinary incontinence (UI) in prostatectomy patients using unstructured clinical notes from Electronic Health Records (EHRs).

**Methods:**

We developed a weakly-supervised natural language processing tool to extract assessments, as recorded in unstructured text notes, of UI before and after radical prostatectomy in a single academic practice across multiple clinicians. Validation was carried out using a subset of patients who completed EPIC-26 surveys before and after surgery. The prevalence of UI as assessed by EHR and EPIC-26 was compared using repeated-measures ANOVA. The agreement of reported UI between EHR and EPIC-26 was evaluated using Cohen’s Kappa coefficient.

**Results:**

A total of 4870 patients and 716 surveys were included. Preoperative prevalence of UI was 12.7 percent. Postoperative prevalence was 71.8 percent at 3 months, 50.2 percent at 6 months and 34.4 and 41.8 at 12 and 24 months, respectively. Similar rates were recorded by physicians in the EHR, particularly for early follow-up. For all time points, the agreement between EPIC-26 and the EHR was moderate (all p < 0.001) and ranged from 86.7 percent agreement at baseline (Kappa = 0.48) to 76.4 percent agreement at 24 months postoperative (Kappa = 0.047).

**Conclusions:**

We have developed a tool to assess documentation of UI after prostatectomy using EHR clinical notes. Our results suggest such a tool can facilitate unbiased measurement of important PCOs using real-word data, which are routinely recorded in EHR unstructured clinician notes. Integrating PCO information into clinical decision support can help guide shared treatment decisions and promote patient-valued care.

## Introduction

Accurate assessment of patient-centered outcomes (PCOs) following therapy for localized prostate cancer is critical in counseling patients regarding treatment selection and improving treatment outcomes. Unfortunately, most data regarding these outcomes are limited to single-institution case series [1], surveys of patients using standardized instruments [2,3], or registry-based observational studies. [4] Of these methods, only patient surveys provide accurate estimations of PCOs, although implementation of surveys can be costly and cumbersome for patients and physicians. In the absence of these data, it is very difficult for physicians to get the feedback necessary to improve their surgical outcomes. Accordingly, efforts to establish guidelines for the selection of prostate cancer treatment remain inconclusive due to insufficient evidence regarding relative benefits and risks of the different treatment options [5,6].

The widespread adoption of electronic health records (EHR) offers opportunities to extract information on treatment-related outcomes from clinicians’ notes routinely recorded as free text in the EHR. The advent of increasingly sophisticated tools, such as natural language processing (NLP), can be used to efficiently identify and extract unstructured data from EHRs, such as outcomes reported by physicians [7]. However, one significant concern is that physicians fail to report PCOs in their notes [8,9].

Based on previous data showing agreement between physician-recorded outcomes [1] and those from objective surveys in men undergoing radical prostatectomy, we hypothesized that outcomes recorded in physicians’ notes would correlate well with those reported by patients using the Expanded Prostate Cancer Index Composite (EPIC). To test this hypothesis, we developed a neural network based Natural Language Processing (NLP) tool to extract physician-reported outcomes from the EHR for urinary incontinence after radical prostatectomy and compared these with patients’ reported outcomes using the EPIC validated instrument [10,11].

## Materials and Methods

### Data Source

We assessed patient records from a tertiary academic medical center accessed under an Institutional Review Board (IRB) approved protocol. EPIC surveys were collected under a separate IRB approved protocol and with informed consent. This health care system has implemented a fully functional EHR system in 2008 (Epic Systems Corporation, Verona Wisconsin). Surveys were linked to patients’ EHRs via unique patient features (first name, last name, date of birth, date of surgery, and surgeon). We linked data from the EHRs with the California Cancer Registry to create a research data warehouse for prostate cancer, described previously [12].

### Study Population

Prostate cancer was identified in the data warehouse using ICD-9/ICD-10 and CPT codes, 2005–2017 [12]. (Supplementary Table 1) Prostate cancer patients undergoing radical prostatectomy were recruited to participate in a prospective evaluation of PCOs, assessed with the EPIC-26 questionnaire at baseline (prior to surgery) and at 3-, 6-, 12- and 24 months after prostatectomy. The questionnaire was administered during or shortly after the clinical visit.

Natural Language Processing (NLP) tool:

We assessed UI for each patient using a weakly-supervised NLP tool that annotates EHR free-text notes for clinical concepts. The tool uses a UI dictionary created by 4 clinicians/nurses (JDB, BIC, MF, DG) which contains 65 unique terms indicative of UI. The notes were first processed using a standard tool of text cleaning methodologies (e.g. stop words and punctuation removal, number to string conversion, and bigram formation using mutual information). To reduce variability in expressing negation and risk, we used the CLEVER dictionary to map synonyms to a standard term list [13]. For instance, [‘no’, ‘absent’, …] are mapped to [NEGEX}] and [‘suspicion’, ‘probable’, …}] mapped to risk [RISK].

We trained a neural word embedding model (Word2Vec) on 528,362 unique pre-processed clinical notes including progress notes, discharge summaries, telephone call notes, and radiology reports. A weighted function of word embedding was used to create a sentence-level vector representation of relevant expressions (containing at least one term from the UI dictionary) extracted from the clinical notes.

Next, we employed the domain-specific dictionaries containing a set of affirmative expressions for UI to build an artificial training set. Sentence vectors were used as input for a logistic regression model, with output being the presence or absence of UI. The classifier was trained on the UI dictionary. Finally, sentence level annotations were aggregated to the note level by majority voting across sentence-level annotations.

### Outcomes and Covariates

Using the NLP tool, we identified affirmed and negated clinical mentions of urinary incontinence/bother from EHRs based on documentation in clinical notes. A gold standard was used to evaluate our tool consisting of 200 records. Using these data, precision and recall metrics were calculated.

In EPIC-26, scores for urinary incontinence/bother were calculated using the previously published methods [14]. To directly compare the EPIC-26 questionnaire and EHR, we created a dichotomous variable from EPIC-26 items. Urinary incontinence (q23: “Over the **past 4 weeks**, how often have you leaked urine?”) and bladder control (q26: “Which of the following best describes your urinary control **during the last 4 weeks**?”) were used to define and assess “Urinary Incontinence”, the outcome of interest.

In detail, using Boolean operators for better explanation, we classified a patient as:

– “**continent**” if he answered: 4 (“*About once a week*”) OR 5 (“*Rarely or never*”) to q23 AND answered 3 (“*Occasional dribbling*”) OR 4 (“*Total control*”) to q26
– “**incontinent**” if he answered: 1 (“*More than once a day*”) OR 2 (“*About once a day*”) OR 3 (“*More than once a week*”) to q23 AND 1 (“*No urinary control whatsoever*”) OR 2 (“*Frequent dribbling*”) OR 3 (“*Occasional dribbling*”) to q26

If a response to q23 or q23 was missing or responses to the two questions were inconsistent, UI was not classified for that survey.

UI derived from these questions was tested for accuracy against diaper use (q27: use of pads or diapers – dichotomous variable that can be answered “yes” or “no”) and bother (q28: classified as “No problem or small problem” vs “moderate or big problem”). This allowed us to check for the internal consistency of the questions answered as far as the Urinary Subscale of the EPIC-26 questionnaire was concerned. Demographic and clinical data were extracted from the EHRs. Charlson’s comorbidity index (CCI) score was used to define the comorbidity severity and categorized into three levels: mild (1–2); moderate (3–4); and severe (≥5) [15]. Gleason grade group was categorized: 1 = low grade; 2–4 = medium grade; 5 = high grade.

### Statistical analysis

Continuous variables were summarized as mean ± standard deviation (SD), categorical variables as frequencies and percentages. Student’s t-test was used to compare mean values between groups. The prevalence of UI at each time point as assessed by EHR and EPIC-26 was compared using repeated-measures ANOVA. The agreement of UI between EHR and EPIC-26 was evaluated using Cohen’s kappa at each time point and rated as fair, moderate, substantial and almost perfect using the cut-offs of 0.21, 0.41, 0.61 and 0.81 defined by Landis and Koch [16].

All statistical analyses were performed using STATA, version 14 and SPSS, version 21. A p-value <0.05 was considered statistically significant.

## Results

**Figure 1:**
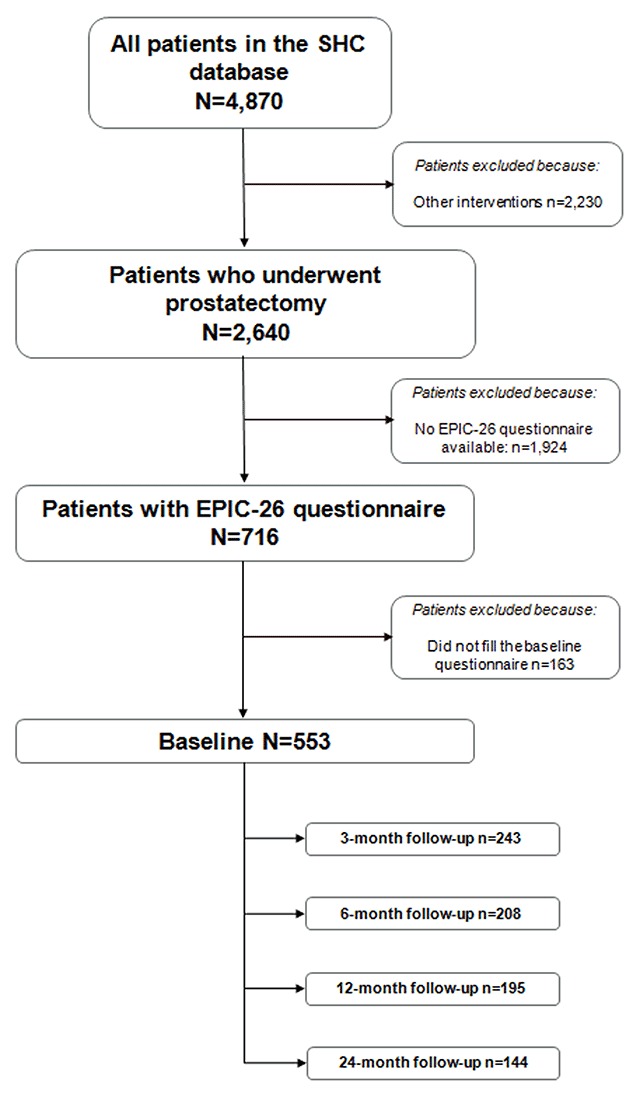
Flowchart for Cohort Selection.

A total of 716 subjects consented to participate in the EPIC-26 study and 553 completed the baseline assessment and had at least 1 assessment of UI in the EHR at baseline or follow-up (Figure 1). The patient characteristics of this subset were compared with those who did not complete the baseline assessment (N = 163) and no significant differences were found, suggesting that patients who completed the EPIC-26 were representative of the overall cohort who underwent surgery (data not shown). We calculated post-hoc power. Given a mean effect f-size difference calculated as 0.07 points for the 4 repeated measures in the total patients, we achieved a power of 0.84, which is considered adequate. Baseline characteristics of the 553 participants are displayed in Table 1.

**Table 1:** Demographic and clinical baseline characteristics.

Demographic characteristics	Mean (±SD) or N (%)

Total	553
Age, mean (SD), y	62.4 (±5.2)
Race	
White/Caucasian	416 (75%)
Black	17 (2.0)
Asian/Pacific Islander	50 (6.6)
Other/Unknown	70 (4.8)
Ethnicity	
Hispanic	22 (3.9%)
Non-Hispanic	501 (90.5%)
Unknown	30 (5.42%)
Marital status	
Married/life partner	469 (84.8%)
Single/Other	84 (15.1%)
English speaking	534 (96.5%)
Insurance status	
Public	251 (45.3%)
Private	276 (50%)
Other	26 (4.7%)
Year of diagnosis	
2005–2010	204 (36.88%)
2011–2017	349 (63.11%)
Clinical characteristics	
Hormone therapy	93 (16.81%)
Chemotherapy	62 (11.21%)
Grade* (Differentiated, Not Otherwise Specified)	
Low Grade	114 (20.61%)
Medium, Intermediate Grade	388 (70.16%)
High Grade	41 (7.41%)
Undefined	10 (1.8%)
Stage, Overall	
1	85 (15.3%)
2	354 (64.01%)
3	10 (1.84%)
4	6 (1.8%)
Unknown	98 (17.72%)
Charlson’s score, mean (SD)	2.98 (±1.95)
Body Mass Index, mean (SD)	26.98 (±7.22)

* As defined by the SEER Program Coding and Staging Manual^20^.

The sensitivity and specificity of UI measured from EPIC-26 with respect to diaper use and urinary bother were greater than 95 percent and 85 percent respectively across all time points (Supplemental Table 2).

**Figure 2:**
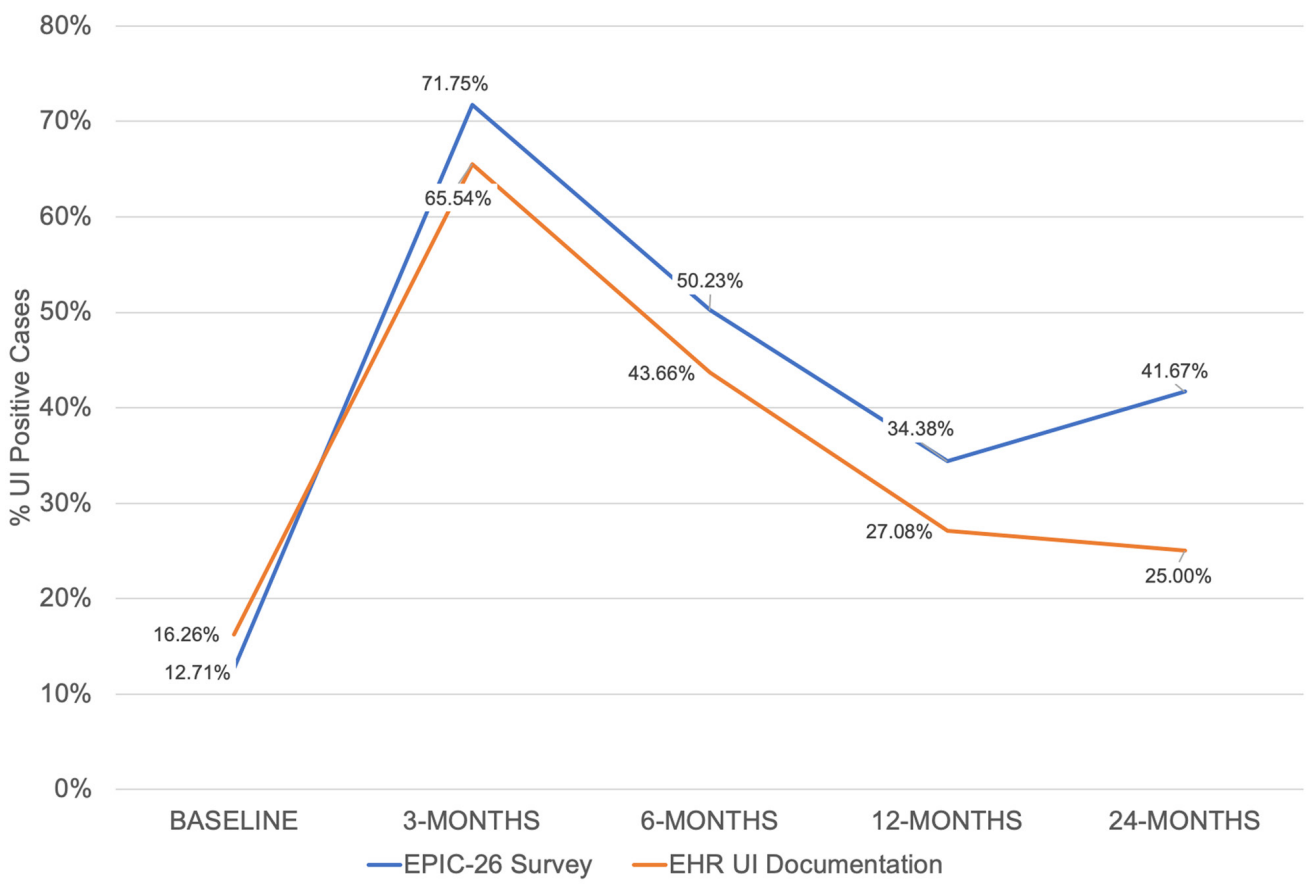
Urinary Incontinence Prevalence from EHR and EPIC-26 at Baseline and Post-Surgery.

Over the course of 24 months postoperative follow-up, the UI rates extracted from clinicians’ notes in the EHR resembled those reported directly by the patients on EPIC-26 (Figure 2). At baseline (pre-operative) UI prevalence derived from EPIC-26 was 12.7 percent and did not diverge from the incontinence rates documented in the EHR (16.3 percent, p = 0.33). Post-operative UI increased to 71.8 percent by 3 months and decreased to 50.2 percent at 6 months in the EPIC-26 questionnaire, with similar but lower rates reported by physicians in the EHR (65.5 percent, p = 0.14 at 3 months; 43.7 percent, p = 0.18 at 6 months). At 12 and 24 months, rates decreased to 34.4 percent and 41.7 percent in the EPIC-26 questionnaire, with significant lower rates of reported incontinence in the EHR (27.1 percent, p = 0.13 and 25.0 percent, p = 0.002, respectively). Kappa values between EPIC-26 and EHR showed moderate agreement at all the timepoints (Table 2), indicating that the similarity of the UI assessments was unlikely to be due to chance alone.

**Table 2:** Observed percentage agreement between EPIC-26 and EHR, expected agreement by chance alone and Cohen’s Kappa.

Urinary incontinence	Observed agreement	Expected agreement	Level of agreement	Cohen’s Kappa	Std. Error	P

**Baseline**	86.7%	75.2%	Moderate	0.48	0.06	<0.0001
**3 months**	77.8%	56.2%	Moderate	0.49	0.06	<0.0001
**6 months**	73.1%	50%	Moderate	0.46	0.07	<0.0001
**12 months**	78.5%	56.8%	Moderate	0.50	0.07	<0.0001
**24 months**	76.4%	55.3%	Moderate	0.47	0.07	<0.0001

Kappa <0 indicates no agreement, 0–0.20 slight, 0.21–0.40 fair, 0.41–0.60 moderate, 0.61–0.80 substantial, and 0.81–1 as almost perfect agreement.

The F-score measure for the UI annotations against an unseen gold standard manually annotated by our board-certified Urology nurse was 0.86 on 200 sentences extracted from the clinical notes.

Finally, the calculated UI scores from EPIC-26 in our study population at baseline and subsequent follow-ups were not statistically different to those reported in several recent studies (Supplemental Table 2).

## Discussion

We developed an information extraction tool that captures clinicians’ documentation of important PCO in prostate cancer patients using routinely recorded unstructured text notes from EHRs. Our tool accurately identified assessments of UI following prostatectomy, and we found that the clinicians’ reporting of UI symptoms is moderately concordant with patients’ reporting using the validated EPIC-26 survey. In addition, our method is highly efficient and can assess over 1 million unstructured clinical notes for a particular outcome in less than 24 hours. In our study, clinicians accurately documented UI in the unstructured narrative text of EHRs and we were able to identify and extract this information using a data-mining tool. This automated method is an important proof of concept that can have a wide variety of applications and is neither limited to UI nor cancer-related outcomes. Advancing the assessment of PCOs to such scalable automated methodologies could have potentially large effects on PCO research in general.

Our findings have several important implications. First, we found a moderate concordance between UI as reported in EPIC-26 with those recorded in the medical record. In other words, the outcomes reported by the patient to their physicians that are subsequently recorded in the medical record appear to reflect the patients’ experience. This moderate degree of correlation was attained despite the presence of several physicians in our practice with unique styles and phrasing in their clinical notes, including free text, smart text, and pull-down menus. Furthermore, the results of UI reported from the EPIC-26 survey in our clinical practice correlate well with several recent reports in diverse patient populations and practice settings which UI was assessed using EPIC-26 surveys after radical prostatectomy [2,3,17]. Our findings are reassuring in light of concerns that patients under-report adverse outcomes to their physicians [8]. In addition, our findings agree with previous reports demonstrating that there is a high degree of concurrence between urinary bother rates collected by patient survey and those recorded by the physician [1].

However, concordance was not perfect, and physicians did under-report UI and at 12- and 24-months follow-up these differences appeared to increase. There are several possible explanations for this discordance. First, physicians and patients are likely to be more focused on UI symptoms immediately following treatment, when patients are in the recovery phase and when incontinence tends to be at its worst. Second, patients may be less likely to report these symptoms at 12 and 24-month follow-up to their surgeon, regardless of their continuing presence. Previous literature suggests that patients may minimize symptoms when discussing with their clinician to avoid subsequent treatment, out of embarrassment, because they have adjusted to them, or to please their care giver [18]. Finally, physicians and patients might fail to report minor degrees of incontinence (drops, post void dribbling, use of a small panty liner) since these have not been considered incontinent in previous surgical series [1]. This work highlights the need for continued assessment and documentation of UI following prostatectomy, particularly for long-term follow-up.

The correlation between PCOs as recorded by EPIC-26 and the NLP method we have developed, while not perfect, opens opportunities for analysis of PCOs in large and diverse EHRs. Our method allows incorporation of diagnostic and procedure codes for extracting outcomes, although these data provide only a component of the information critical to assessing outcomes. Previous studies have shown that assessment of outcomes using only diagnostic and procedure codes for UI are incomplete and often inaccurate [7]. By adding critical information found in the narrative, free text comments from the physicians, accuracy levels improve dramatically. Our ability to extract these outcomes opens the possibility to efficiently and accurately assess PCOs, at least with regard to UI. Our method can also be deployed in many clinical practice contexts and in diverse EHR systems, particularly since our algorithm is open source. The extraction of PCOs from clinical notes can also address issues of ascertainment bias associated with patient surveys. This opens the possibility of assessing patient-centered outcomes at the practice level, provider level, and population level.

While our method assesses urinary bother after prostatectomy, it should be regarded as a surrogate for urinary outcomes and not intended to replace assessments of PCOs of urinary symptoms obtained by well-designed and validated instruments such as EPIC-26. Data extracted from EHR’s can play an important role in benchmarking outcomes in practice settings where it is difficult or impossible to obtain surveys capturing PCOs on every patient seen in routine clinical care. In the future, capture of patient centered outcome data will be used to drive health care improvement across the health care system, but must be made available at the practice level. It is unclear whether this will be done using automated methods, such as those we have developed for urinary bother, or though structured notes in the EHR, which will be expensive to deploy and potentially increase the documentation burden on providers.

Our study does have limitations. Development of tools for assessing specific PCOs depends on assembling vocabularies related to the outcome of interest. This can be cumbersome and could be confounded when deployed in practice settings where different or idiosyncratic terminologies are used. However, we have developed and disseminated electronic phenotypes that capture these vocabularies for the purpose of external validation [19]. In our experience, external validation has proven challenging since other health care systems with EHRs have been reluctant to run the algorithms because of limited information technology support internally, patient privacy concerns, or concerns about comparing PCO between institutions. As more health care systems utilize these vocabularies, algorithm modifications can improve accuracy and allow for more rigorous independent validation. Second, our method does not capture the degree or type of urinary incontinence, and the numbers we report encompass a range of urinary bother and types, similar to results from previous survey-based reports [2,3,17]. We are continuing to refine our algorithms to extract information on the type and degree of urinary bother to make it more broadly applicable. Third, while our work demonstrates that our algorithm works well for UI, it is likely to be difficult to capture more complex outcomes such as erectile dysfunction, which are more subjective. Our method relies on physician documentation of patient outcomes. While the outcomes reported in our health care system track well with those reported by the patient, this might not be true in other practice settings. However, as clinician documentation is becoming a viable data component of clinical analytics, it is likely that more accurate and complete text documentation from clinicians will become expected in the future. Finally, we collapsed responses using the EPIC-26 instructions. Transforming two categorical variables into dichotomous ones may have reduced the power of our analysis, but this was the only way to obtain a direct comparison between the responses to EPIC-26 and the outcomes as assessed in the EHR.

In this study, we present new methods to advance patient-centered outcomes research – leveraging the vast amount of routinely collected retrospective data on patient-centered outcomes in EHRs. The information we found embedded in the unstructured clinical narrative text related to urinary incontinence was comprehensive and concordant with a validated instrument used to capture outcomes after prostate cancer treatment, the EPIC-26 survey. This methodology opens avenues to more thorough examination PCO, providing power to support predictive analytics and subpopulation analyses. Bringing such evidence to the point of care through clinical decision support tools will allow patients to: 1) better understand the risks of PCO across treatment pathways; 2) allow comparison of PCO across providers and systems; and 3) evaluate personalized risk of PCOs across patients with similar demographic and clinical characteristics. This study demonstrates how we can use unstructured text generated in routine clinical care to assess outcomes not routinely captured as structured data and to potentially guide treatment decisions.

## Conclusion

We have developed a data-mining tool for extracting urinary incontinence outcomes after prostate cancer surgery that displays a moderate degree of agreement with patient reported outcomes using a validated instrument. Future work will expand to include assessment of additional quality metrics and patient centered outcomes. In addition, we will validate the performance of the algorithms we have developed in independent EHRs. This method opens significant opportunities for development of datasets for addressing patient and disease centered research across health care systems and populations.

## Additional Files

The additional files for this article can be found as follows:

10.5334/egems.297.s1 Supplemental Table 1. Diagnostic and Procedure codes used to identify prostate cancer patients.

10.5334/egems.297.s2 Supplemental Table 2. Sensitivity and specificity of UI in the EPIC-26 Questionnaire.

10.5334/egems.297.s3 Supplemental Table 3. Urinary incontinence scores from EPIC-26 at different times and comparison with previously conducted studies.
