# Supplementary material for: Extracting Patient-Centered Outcomes from Clinical Notes in Electronic Health Records: Assessment of Urinary Incontinence After Radical Prostatectomy
Source: EGEMS (Wash DC). 2019 Aug 20;7(1):43. doi: 10.5334/egems.297 (PMC6706996; doi:10.5334/egems.297)
Supplement: Supplemental Table 1. — Diagnostic and Procedure codes used to identify prostate cancer patients. [file egems-7-1-297-s1.pdf]

Supplemental Table 1. Diagnostic and Procedure codes used to identify prostate cancer patients.

Variable	Code Type	Code
**Prostate Cancer**	ICD-9	185
	ICD-10	C61
**Prostatectomy**	CPT	55801-55845, 55866
	ICD-9	60.0-60.6
	ICD-10	60.61-61.69
