# Supplementary material for: Extracting Patient-Centered Outcomes from Clinical Notes in Electronic Health Records: Assessment of Urinary Incontinence After Radical Prostatectomy
Source: EGEMS (Wash DC). 2019 Aug 20;7(1):43. doi: 10.5334/egems.297 (PMC6706996; doi:10.5334/egems.297)
Supplement: Supplemental Table 2. — Sensitivity and specificity of UI in the EPIC-26 Questionnaire. [file egems-7-1-297-s2.pdf]

Supplemental Table 2: Sensitivity and specificity of UI in the EPIC-26 Questionnaire*

UI definition	Sensitivity**	Specificity**
**Baseline**	96.82% (94.33 – 97.27)	100% (99.12 – 100)
**3 months**	97.01% (92.07 – 98.98)	88.13% (83.43 – 93.55)
**6 months**	95.11% (92.36 – 96.94)	87.23% (84.77 – 90.75)
**12 months**	100% (98.43 – 100)	93.77% (90.99 – 96.55)
**24 months**	96.22% (91.62 – 99.01)	95.41% (92.43 – 99.18)

*UI (as dichotomous) in q23 (incontinence) and q26 (control) as compared to q27 (diaper use) and q28 (urinary bother)

** Percentage and 95% confidence intervals
