# Supplementary material for: Extracting Patient-Centered Outcomes from Clinical Notes in Electronic Health Records: Assessment of Urinary Incontinence After Radical Prostatectomy
Source: EGEMS (Wash DC). 2019 Aug 20;7(1):43. doi: 10.5334/egems.297 (PMC6706996; doi:10.5334/egems.297)
Supplement: Supplemental Table 3. — Urinary incontinence scores from EPIC-26 at different times and comparison with previously conducted studies. [file egems-7-1-297-s3.pdf]

Supplemental Table 3: Urinary incontinence scores from EPIC-26 at different times and comparison with previously conducted studies.

Follow up	Gori et al. 2017 (This study) N=301	Donovan et al. 2016[19] N=553	Chen et al. 2017[3] N=462	Barocas et al. 2017[2] N=1523	p-value
**baseline**	92.8 (1.1)	92.8 (3.4)	90.3 (2.5)	86.7 (1.2)	n.s.
**3 months**	52.1 (2.9)	-	54.8 (5.3) – 2 months	-	n.s.
**6 months**	68.9 (2.7)	67.4 (5.3)	-	65.4 (1.6)	n.s.
**12 months**	74.5 (2.9)	76.5 (4.9)	67.7 (5.4)	70.7 (1.5)	n.s.
**24 months**	75.2 (2.9)	80.3 (4.6)	67 (4.8)	71.8 (1.4) - 3 years	n.s.
